# Mammalian phagophores with finger-like shapes emerge from recycling endosomes

**DOI:** 10.1080/15548627.2023.2293439

**Published:** 2023-12-14

**Authors:** Claudia Puri, David C Rubinsztein

**Affiliations:** a Department of Medical Genetics, Cambridge Institute for Medical Research, Cambridge, UK; b UK Dementia Research Institute, University of Cambridge, Cambridge, UK

**Keywords:** Autophagy, ESCRT, phagophore, RAB11, recycling endosome

## Abstract

Autophagosomes are double-membraned vesicles that engulf cytoplasmic contents, which are ultimately degraded after autophagosome-lysosome fusion. The prevailing view, largely inferred from EM-based studies, was that mammalian autophagosomes evolved from disc-shaped precursors that invaginated and then were closed at the single opening. Many site(s) of origin of these precursors have been proposed. Using superresolution structured illumination microscopy and electron microscopy, we find that mammalian autophagosomes derive from finger-like outgrowths from the recycling endosome. These “fingers” survey a large cell volume and then close into a “fist” and the openings are sealed in an ESCRT-dependent fashion, while the precursors are still attached to the recycling endosome. We call this transient recycling endosome-attached, closed, autophagic structure an “autophago-dome”. DNM2-dependent scission of the autophago-dome from the recycling endosomes liberates free autophagosomes from this compartment. These data reveal unexpected morphologies of autophagosome precursors and raise new questions about the control of this process.

How mammalian cells form double-membraned autophagosomes has been a fundamental question in the field. Models have considered that autophagosomes derive predominantly from one organelle or that they form *de novo* with membranous input from multiple sources (or a hybrid model including both possibilities). Based on electron microscopy, EM-tomography reconstructions and theoretical modeling, mammalian autophagosomes have been presumed to derive from disc-shaped precursors that invaginate to form phagophores which have been almost universally portrayed as round, vase-shaped structures with single openings (Figure 1A). However, phagophore morphologies have been largely inferred from 2-D EM studies and one could obtain C-shaped 2-dimensional structures if they had more than one opening – such structures could result if more complex multi-aperture organelles were sliced in an orientation that only went through one opening. If one were to approach EM data with the expectation that phagophores were vase-shaped structures, then one would ignore or may not be able to interpret intermediates where there were more than one opening captured in the slice being analyzed. Fixed cell analyses may also overlook unexpected transient intermediates with low steady-state abundances.

**Figure 1.**
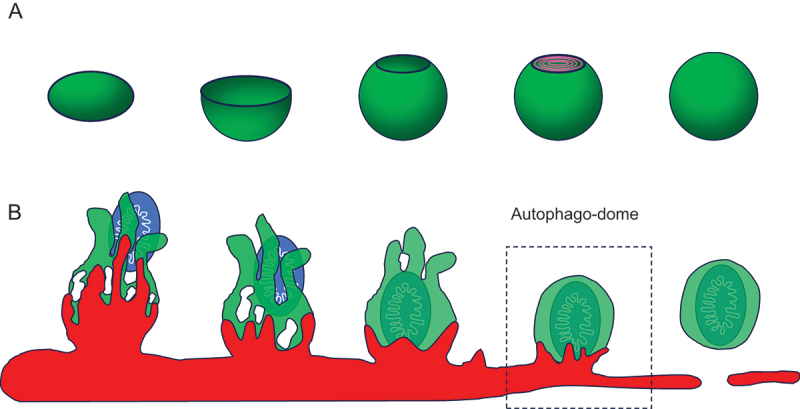
(A) schematic illustration of the conventional view of phagophore-autophagosome morphological transitions. (B) schematic representation of the phagophore intermediates we observe with superresolution microscopy. The autophago-dome is an intermediate structure where the closed autophagosome with its engulfed contents is still attached to the recycling endosome. This is then released in a DNM2-dependent manner.

Our previous work has shown that LC3-family membrane conjugation, a defining step in autophagosome biogenesis, along with the location of much of the machinery required for this process, occurs on RAB11A-positive recycling endosomes. Yoshimori and colleagues showed that the membranes conjugated with LC3-family members are specified by the localization of the ATG16L1-containing E3-like complex that mediates this process. Tooze and colleagues demonstrated that ATG16L1 localization is, in turn, determined by its binding partner WIPI2 – thus, WIPI2 location determines which membranes are conjugated by LC3. We found that WIPI2 localization to autophagosome precursor membranes (and autophagosome biogenesis) is determined not only by its binding to PtdIns3P but also by co-incident binding to RAB11A, a recycling endosome marker protein. These data argued that recycling endosomes constitute the primary platform on which autophagosomes form. (There are likely additional inputs into autophagosomes from other sources like the ER and the secretory pathway.)

We then showed that DNM2-dependent scission releases autophagosome precursors from recycling endosomes. When this is perturbed with various DNM2 mutants, sealed autophagosomes accumulate on the RAB11A compartment. Thus, we tested if autophagosome closure was a prerequisite for release from the recycling endosomes. We validated data from Wang’s lab showing that ESCRT function is required for autophagosome sealing and showed that if we inhibit ESCRT function, autophagosome release from recycling endosomes is compromised.

These data also suggested that we can block this closure and test if autophagosomes derive from vase-shaped precursors with single apertures. When we examined control cells with endogenous LC3B staining with superresolution structured illumination microscopy, we observed an overwhelming predominance of round structures (which we presumed to be closed autophagosomes), very occasional finger-like structures with multiple openings attached to the RAB11A compartment, and never saw structures with single-openings that looked like textbook phagophores [1]. These finger-shaped structures are likely authentic autophagosome precursors, as they predominate when we block autophagosome closure. Importantly, a VPS4A dominant-negative mutation which is predicted to get stuck at its sites of action localized at multiple openings on individual phagophores. We observe the same situation in HeLa, SH-SY5Y (neuroblastoma), U-2 OS (osteosarcoma), and H4 (neuroglioma) cell lines, and when we use antibodies to either LC3A or WIPI2. Similar (and overlapping) finger-like morphologies of phagophores are seen when we use a lipid dye recognizing autophagosomes, DALGreen, or when we mark autophagosomes with endocytosed transferrin that we chase into recycling endosomes. Interestingly, the morphologies of some autophagosome precursor-like structures that we observe in ESCRT-compromised cells by superresolution microscopy are compatible with those seen using whole-mount electron microscopy.

We further confirmed that these structures are phagophores by simultaneously labeling autophagosomes with LC3B or DALGreen and visualizing macroautophagy/autophagy substrates, like SQSTM1/p62 or mitochondria, inside the finger-like structures with the substrates being visible in the gaps between the “fingers”.

Live-imaging structured illumination microscopy confirmed that autophagosome precursors emerge from the recycling endosome and start as finger-like structures which survey a large volume of cytoplasm to capture substrates. The fingers then close down (analogous to forming a fist with a smaller size compared to the volume occupied by the fingers) and the apertures are sealeded in an ESCRT-dependent fashion. When ESCRT function is compromised the structures remain finger-like and attached to the recycling endosome, compared to the control cells where the autophagosomes are freed from their recycling endosome origin platform (Figure 1B).

The structures we have described may have been missed in previous EM-related studies in mammalian cells, as the identification of early autophagic structures relies on molecular markers that define these initial events that can be labeled with fluorescent tags or immunolabelled with specific antibodies. These organelles can be easily identified using our fluorescent super-resolution techniques in whole cells. Such precursors may also have been overlooked in EM studies due to their low steady-state abundance, while the superresolution approach makes it possible to survey all the labeled structures in a section of a given cell. Furthermore, the phagophores we have observed occupy a large volume (500–1500 nm across), that may have been very difficult to capture in previous electron tomography studies, as these would have necessitated joining together multiple serial tomograms each capturing smaller volumes. Such reconstructions of entire early autophagosomal structures would still not enable confident visualization of finger-like structures within thick sections containing a plethora of other endocytic and biosynthetic vesicles and tubules using only morphological criteria in the absence of molecular markers.

In summary, our data reveal that autophagosomes derive from finger-like LC3-positive projections from the recycling endosome. These engulf cytoplasmic contents and then close to form an autophago-dome, still attached to the recycling endosome. The closed autophagosomes are then released from the recycling endosomes by DNM2-dependent scission. These unexpected structures may enable more efficient substrate capture by surveying a larger cytoplasmic volume. The persistence of these structures may contribute to pathologies seen in diseases caused by defects in the ESCRT machinery, including forms of ALS, frontotemporal dementia and multisystem disease including structural brain abnormalities, severe neurodevelopmental delay, cataracts, growth impairment, and anemia.
